# Elusive Role of the CD94/NKG2C NK Cell Receptor in the Response to Cytomegalovirus: Novel Experimental Observations in a Reporter Cell System

**DOI:** 10.3389/fimmu.2017.01317

**Published:** 2017-10-24

**Authors:** Aldi Pupuleku, Marcel Costa-García, Domènec Farré, Hartmut Hengel, Ana Angulo, Aura Muntasell, Miguel López-Botet

**Affiliations:** ^1^Department of Experimental and Health Sciences, University Pompeu Fabra, Barcelona, Spain; ^2^Immunology Unit, Department of Biomedical Sciences, Medical School, University of Barcelona, Barcelona, Spain; ^3^Institute of Virology, Albert Ludwigs University of Freiburg, Freiburg, Germany; ^4^Faculty of Medicine, Albert Ludwigs University of Freiburg, Freiburg, Germany; ^5^Institut d’Investigacions Biomèdiques August Pi i Sunyer, Barcelona, Spain; ^6^Hospital del Mar Medical Research Institute (IMIM), Barcelona, Spain

**Keywords:** human, natural killer cell, cytomegalovirus, CD94, NKG2C, HLA-E, UL40

## Abstract

Human cytomegalovirus (HCMV) infection promotes the differentiation and persistent expansion of a mature NK cell subset, which displays high surface levels of the activating CD94/NKG2C NK cell receptor, together with additional distinctive phenotypic and functional features. The mechanisms underlying the development of adaptive NK cells remain uncertain but some observations support the involvement of a cognate interaction of CD94/NKG2C with ligand(s) displayed by HCMV-infected cells. To approach this issue, the heterodimer and its adaptor (DAP12) were expressed in the human Jurkat leukemia T cell line; signaling was detected by transfection of a reporter plasmid encoding for Luciferase (Luc) under NFAT/AP1-dependent control. Engagement of the receptor by solid-phase bound CD94- or NKG2C-specific monoclonal antibodies (mAbs) triggered Luc expression. Moreover, reporter activation was detectable upon interaction with HLA-E+ 721.221 (.221-AEH) cells, as well as with 721.221 cells incubated with synthetic peptides, which stabilized surface expression of endogenous HLA-E; the response was specifically antagonized by soluble NKG2C- and HLA-E-specific mAbs. By contrast, activation of Jurkat-NKG2C+ was undetectable upon interaction with Human Fetal Foreskin Fibroblasts (HFFF) infected with HCMV laboratory strains (i.e., AD169, Towne), regardless of their differential ability to preserve surface HLA-E expression. On the other hand, infection with two clinical isolates or with the endotheliotropic TB40/E strain triggered Jurkat-NKG2C+ activation; yet, this response was not inhibited by blocking mAbs and was independent of CD94/NKG2C expression. The results are discussed in the framework of previous observations supporting the hypothetical existence of specific ligand(s) for CD94/NKG2C in HCMV-infected cells.

## Introduction

Inhibitory receptors specific for MHC class I molecules with immunoreceptor tyrosine-based inhibitory motifs play a key role in preventing NK cell responses against normal autologous cells. This function is mainly fulfilled by members of the human killer-cell immunoglobulin-like receptor (KIR) family, which recognize sets of classical HLA class I (HLA-I) molecules, and by the CD94/NKG2A lectin-like heterodimer specific for HLA-E. Conversely, other KIRs and CD94/NKG2C, which display a lower affinity for HLA-I ligands trigger protein tyrosine kinase pathways through DAP12, an adaptor with immunoreceptor tyrosine-based activation motifs. Similar inhibitory and activating NK cell receptors (NKR) have been identified among the murine Ly49 and NKG2 lectin-like receptor families (1, 2). The hypothesis that MHC-specific activating NKR may contribute to the innate response against pathogens was supported by the evidence that Ly49H specifically interacts with the MHC class I-related murine cytomegalovirus glycoprotein m157, triggering NK cell effector functions and the development of a memory-like response that confers resistance against the viral infection in some mice strains (3–5).

With this remarkable exception, no formal proof has been thus far obtained supporting the involvement of other activating KIR, NKG2, or Ly49 receptors in direct recognition of pathogen molecules (6). In this regard, human cytomegalovirus (HCMV) infection has been shown to promote the differentiation and persistent expansion of a mature NK cell subset, which displays high surface levels of the activating CD94/NKG2C NKR (NKG2C^bright^), together with additional distinctive phenotypic and functional features (7–12). The magnitude of such adaptive NK cell subset redistribution appears variable in healthy blood donors, being undetectable in some HCMV+ individuals. This NK cell response pattern has been as well observed following active HCMV infection in newborns and immunocompromised patients (13–17). Although expansions of NKG2C+ NK cells have been reported in the context of other infections (18–21), the effect appears restricted to individuals coinfected by HCMV, thus suggesting that it is specifically induced by this herpes virus, being potentially amplified along the immune response to other pathogens.

The mechanisms underlying differentiation and expansion of NKG2C^bright^ NK cells remain uncertain. Engagement of CD94/NKG2C by specific monoclonal antibodies (mAbs) or HLA-E, expressed in the 721.221 (.221) HLA-I defective cell line, triggered NKG2C+ NK-cell effector functions and proliferation in response to IL-2 or IL-15, strongly suggesting that the receptor might play a direct role in the response to HCMV infection (22, 23). *In vitro* proliferation of NKG2C+ cells was observed coculturing PBMCs or purified NK cells from some HCMV+ donors with HCMV-infected fibroblasts. The response required the participation of cytokines (i.e., IL-12, IL-15) and was antagonized by anti-CD94 (22), -NKG2C, or -HLA-E mAbs (23). These observations supported the hypothesis of an instructive process driven by a cognate interaction of the CD94/NKG2C receptor with ligand(s) displayed by HCMV-infected cells (24). Paradoxically, no formal evidence has been obtained supporting an active role of the CD94/NKG2C receptor in triggering *in vitro* NK cell effector functions against HCMV-infected cells, suggesting that NKG2C-mediated NK cell activation might be hampered by viral immune evasion mechanism(s) (25). By contrast, antibody-dependent stimulation *via* CD16 (FcγR-IIIA) efficiently activates adaptive NKG2C+ NK cells to mediate specific cytotoxicity, cytokine production, and proliferation in response to HCMV- and other virus-infected cells (26–29). CD2 has been shown to play an important co-stimulatory role in antibody-dependent activation of NKG2C+ cells (30, 31). Recently, increased baseline proportions of adaptive NKG2C+ NK cells in kidney transplant recipients have been directly related with a reduced incidence of posttransplant HCMV infection (32), suggesting that they may play a role in antiviral defense, involving CD94/NKG2C and/or CD16-dependent activation (33).

Previous reports revealed that binding of HLA-E to a peptide from the HCMV UL40 leader sequence preserves its expression in infected cells, engaging the CD94/NKG2A inhibitory receptor (34, 35). On the other hand, viral MHC class I-modulating molecules (i.e., US2-US11) were shown to play a prevalent role in governing the response of NK cells against infected targets (36).

In the present study, we approached the identification of putative ligand(s) for CD94/NKG2C in HCMV-infected cells, reducing the complexity of NK cell-infected target interactions. To this end, both receptor subunits and DAP12 were stably expressed in the human Jurkat leukemia T cell line. Signaling was detected by transient transfection of a reporter plasmid encoding for Luciferase (Luc) under NFAT/AP1-dependent control. Our results are discussed in the hypothetical framework on the development of adaptive NKG2C+ cells in response to HCMV.

## Materials and Methods

### mAbs and Flow Cytometry Analysis

Flow cytometry was performed using mAbs specific for the following surface molecules: anti-NKG2C-PE (clone 134591) R&D Systems (Minneapolis, MN, USA), anti-HLA-I-APC (clone HP-1F7) generated in our laboratory and conjugated by Immunostep (Salamanca, Spain). The following indirect antibodies were used as purified or culture supernatants: anti-HLA-E (clone 3D12) provided by Dr. D. E. Geraghty (Fred Hutchinson Cancer Research Centre, Seattle, WA, USA), anti-CD3 (clone SpvT3B); anti-NKG2A (clone Z199), anti-NKG2D (clone BAT221), anti-NKp46 (clone Bab281), anti-NKp30 (clone AZ20), anti-DNAM1 (clone F22), anti-CD16 (KD1) provided by Dr. A. Moretta (University of Genova), and Dr. D. Pende (National Institute for Cancer Research, Genova); anti-LFA1 (clone TS/18), anti-ICAM1 (clone HU5/3) provided by Dr. F. Sánchez-Madrid (Hospital Univ. de la Princesa, Madrid); anti-KIR3DL1 (clone DX9) provided by Dr. L. Lanier (University of California San Francisco, CA, USA); anti-KIR2DL2/S2/L3 (clone CH-L) provided by Dr. S. Ferrini (National Institute for Cancer Research, Genova, Italy); anti-KIR3DL1/3DL2/2DS4/2DS5/2DS2/3DS1 (clone 5.133), provided by Dr. M. Colonna (University of Saint Louis, MO, USA). Anti-CD94 (clone HP-3B1), anti-ILT2 (LILRB1, LIR1) (clone HP-F1), anti-CD2 (clone MAR206), anti-KIR2DL1 (clone HP-DM1), anti-KIR2DL1/2DS1/2DS3/2DS5 (clone HP-MA4), anti-KIR2DL5 (clone UP-R1), and anti-KIR2DL1/S1/S4 (clone HP-3E4) were produced in our laboratory.

Briefly, cells were pretreated with human IgG (10 µg/ml) to block Fc receptors, incubated with individual NKR-specific mAbs for 30 min, washed, and further incubated with a secondary PE-tagged F(ab’)2 rabbit anti-mouse Ig (The Jackson Immunoresearch, West Grove PA, USA); anti-myc mAb (9E10, IgG1) was used as negative control. Data were acquired on FACSCalibur flow cytometer (BD Biosciences) and processed using FlowJo software (TreeStar, OR, USA).

### Cell Lines and Culture Conditions

The Jurkat leukemia T cell line and its transfectants were grown in RPMI-1640 medium (Gibco, Grand Island, New York, NY, USA) supplemented with 10% heat-inactivated Fetal Bovine Serum (Gibco), 100 U/ml penicillin, and 100 µg/ml streptomycin (Gibco), termed as complete medium. Jurkat-CD94+ cells were kindly provided by Dr. Lewis Lanier, obtained as previously described (37) and cultured in complete medium with G418 (1 mg/ml) (InvivoGen, San Diego, CA, USA).

The 721.221 (.221) HLA-I-deficient EBV-transformed B lymphoblastoid cell line and its transfectant .221-AEH (kindly provided by Dr. D. E. Geraghty, Fred Hutchinson Cancer Research Centre, Seattle, WA, USA) were cultured in RPMI-1640 complete medium .221-AEH cells were generated by stable transfection of .221 cells with a construct in which the leader sequence of the HLA-E*0101 allele was replaced by that of HLA-A2 and were selected in the presence of 300 µg/ml hygromycin B (Invitrogen, Carlsbad, CA, USA) (38).

Synthetic leader sequence peptides from HLA-G (VMAPRTLFL) or the AD169 UL40 viral protein (VMAPRTLIL) were purchased from CRG-UPF proteomic core facility (Parc de Recerca Biomèdica de Barcelona, Spain). As described (39), to stabilize HLA-E surface expression, HLA-Ia-defective 721.221 cells were incubated overnight with peptides (10 mM) at 26°C; HLA-E surface expression was monitored before and after incubation with peptides by flow cytometry.

Human Fetal Foreskin Fibroblast (HFFF) cells provided by Prof. John Trowsdale (University of Cambridge, UK), and the human lung fibroblast cell line MRC-5 provided by Dr. A. Angulo, were maintained in Dulbecco modified essential medium (DMEM) (Gibco) supplemented with 10% FBS, penicillin, and streptomycin.

### Generation of Jurkat-NKG2C+ Reporter Cells

Jurkat-NKG2C+ cells were established using a retroviral expression system to stably express DAP12 and NKG2C proteins in Jurkat-CD94+ cells. DAP12 and NKG2C cDNA constructs were subcloned from pJFE14 expression vector to pBABE-puro retroviral vector using XbaI-EcoRI (DAP12) and BamHI-EcoRI (NKG2C) restriction sites. As described (40), the retroviral constructs were individually transfected using the non-modified polyethyleneimine reagent (PEI, Sigma-Aldrich, St. Louis, MO, USA) into the helper-virus free amphotropic producer cell line Phoenix-A, a derivative of the human embryonic kidney cell line 293T (provided by Dr. Ramon Gimeno, IMIM, Barcelona, Spain). At 48, 72, and 96 h post-transfection, supernatants containing retroviral particles of DAP12 and NKG2C were collected, filtered with 45 µm filter (Millipore, Billerica, MA, USA), and centrifuged with Beckman SW28 rotor at 25,000 rpm for 90 min at 4°C. Pelleted virus were resuspended in 1 ml of RPMI medium and used to transduce Jurkat-CD94+ cells. To this end, 500,000 cells were plated (48 well/plates) in 1 ml mixed concentrated retroviral medium (0.5 ml DAP12 and 0.5 ml NKG2C) in the presence of 8 µg/ml polybrene (Sigma-Aldrich) and spinned for 90 min at 930 *g*. Cells were supplemented with fresh medium at 6 h post-transduction and selected with 1.5 µg/ml puromycin (Sigma-Aldrich) for 48 h; subsequently, cells positive for NKG2C surface expression were sorted (Influx Cell sorter, BD Bioscience), cloned by limiting dilution and expanded.

### HCMV Preparations and Infection of Human Fetal Foreskin Fibroblasts (HFFF)

This work was carried out in an authorized UPF p2-level biohazard facility, in compliance with the official requirements for CMV manipulation. Stocks of concentrated HCMV strains AD169 and Towne, provided by Dr. A. Angulo, TB40/E, provided by Dr. C. Sinzger (Institute for Medical Virology, University of Tübingen), and two HCMV clinical isolates: UL1271 (41) and #119, provided by Dr. H. Hengel, were prepared as follows. Almost confluent MRC-5 fibroblasts were infected at low multiplicity of infection (MOI) and supernatants were recovered when maximum cytopathic effect was reached (7–10 days) followed by clearing of cellular debris by centrifugation at 1,750 *g* for 10 min (42). Thereafter, the virus was concentrated for 3 h by centrifugation at 29,000 *g* at 15°C. Pelleted virus was resuspended in serum-free Dulbecco medium, stored at −80°C, and titrated by standard plaque assays.

Human fetal foreskin fibroblasts were seeded in 48-well plates 2 days prior infection at 4 × 10^4^ cells/well. Confluent cells were incubated alone (mock) or with different viral strains at MOI of 10. After 2 h of absorption at 37°C, cells were washed twice with PBS and then fresh DMEM medium was added. Depending on the experimental design, HFFF cells were washed again at 24, 48, or 72 h postinfection. The infection rate was assessed by monitoring expression of the IE1 protein by indirect immunofluorescence with mAb MAB810R (clone 8B1.2) (Millipore) and Alexa Fluor 488-Labeled F(ab’)_2_ goat anti-mouse secondary (Invitrogen, Carlsbad, CA, USA). Alternatively, infected cells were indirectly identified assessing down-modulationof surface HLA-I expression at 72 h postinfection.

### Preparation and Activation of CD94-NKG2C+ Reporter Cells

Human cytomegalovirus-infected HFFFs were cocultured with Jurkat-NKG2C+ cells previously transfected with a reporter plasmid encoding Luciferase (Luc) under the control of NFAT/AP1 promoter (3X NFAT/AP1-Luc) generated as described (43) and provided by Dr. Jose Aramburu (Universitat Pompeu Fabra, Barcelona, Spain). Transfection of Jurkat cells was carried out using the Neon Transfection System (Thermo Fisher Scientific, Waltham, MA, USA) following the protocol provided by the manufacturer. Luc-transfected Jurkat cells were cocultured with the different targets for 18–24 h. After coculture, cells were collected, lysed, and Luc activity was measured using Promega Luciferase Assay system (Promega, Madison, WI, USA). The data were normalized referring the specific luminescence counts to those of non-treated (NT) Jurkat-NKG2C+ cells and are represented as fold-change induction. Stimulation of Jurkat with plate-bound anti-CD3, -CD94, -NKG2C, or co-culture with 721.221 and .221-AEH cells were used as controls. To verify the involvement of NFAT/AP1 in the reporter activation, experiments were carried out in the presence of 1 µM FK506 calcineurin inhibitor (Sigma-Aldrich), pretreating Jurkat-NKG2C+ cells with the drug for 2 h. For antibody-blocking assays, Jurkat-NKG2C+ cells or infected HFFF cells were, respectively, preincubated for 2 h with anti-NKG2C (clone 134522, R&D Systems) or anti-HLA-E (clone 3D12) mAbs (5 µg/ml) prior to coculture; anti-CD94 F(ab’)_2_ fragments obtained from the HP-3B1 clone (37) were employed in some experiments.

### Statistical Analysis

Jurkat-NKG2C+ cell activation, assessed by induction of Luc activity in response to control stimuli (i.e., anti-NKG2C or .221-AEH cells) in different experiments (*n* = 15), was verified to follow a normal distribution applying the conventional Shapiro–Wilk test. Statistical analysis of the results was carried out applying the Student’s *t*-test.

## Results

### Generation and Phenotypic Characterization of a Human CD94/NKG2C+ Reporter T Cell Line

**Figure 1:**
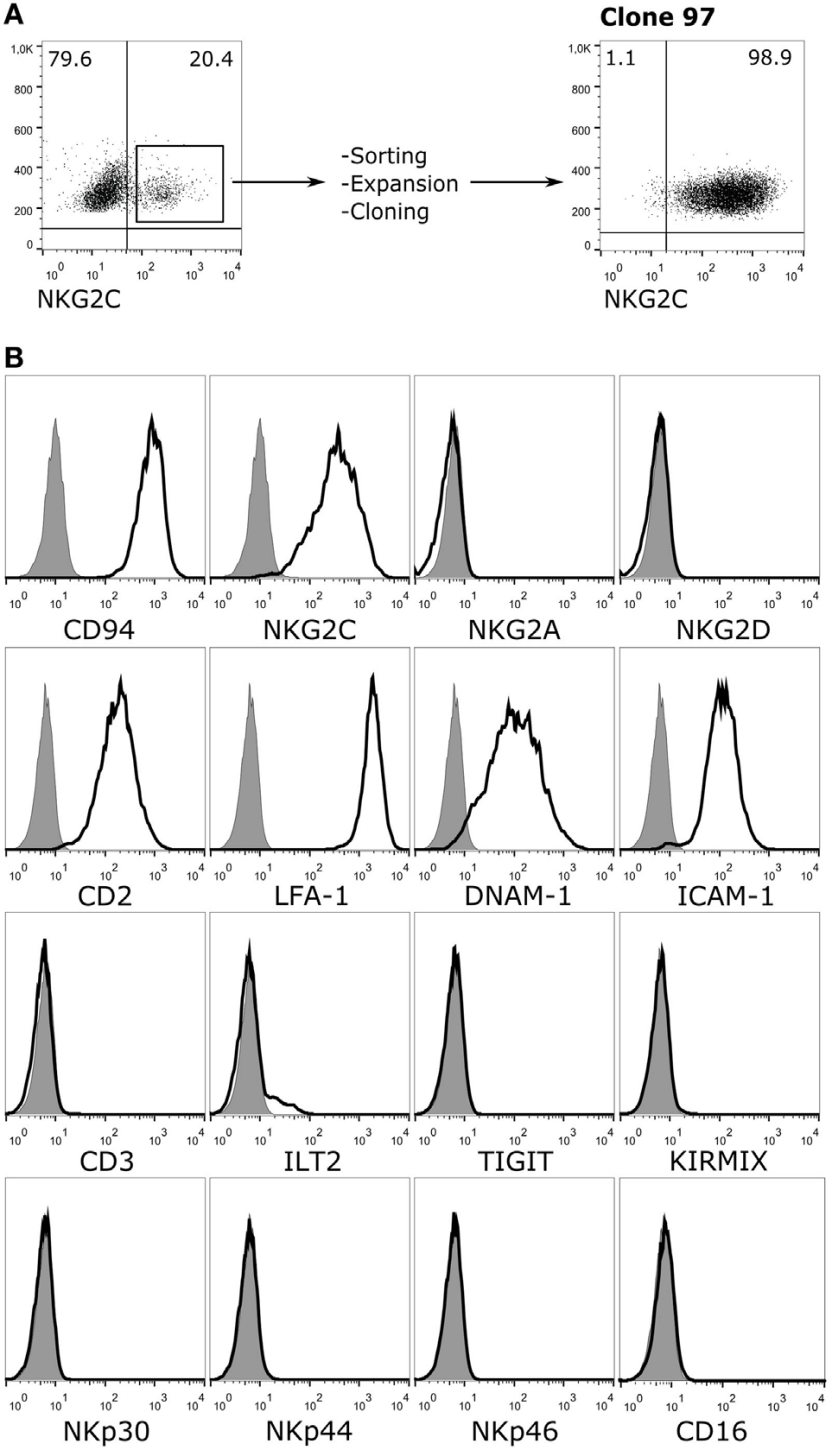
Phenotypic characterization of Jurkat-NKG2C+ reporter cells. **(A)** Jurkat-CD94+ cells were transduced with retroviral particles carrying the sequences encoding for NKG2C and DAP12 as described in Section “Materials and Methods.” After sorting, expansion, and limiting dilution, clone 97, which expressed high levels of the CD94/NKG2C heterodimer was selected. **(B)** Jurkat-NKG2C+ cells were stained for the indicated receptors and analyzed by flow cytometry. KIRMIX corresponds to a mixture of the following monoclonal antibodies: HP-MA4 (KIR2DL1/2DS1/2DS3/2DS5), CHL (KIR2DL2/2DL3/2DS2), 5.133 (KIR3DL1/3DL2/2DS4/2DS5/2DS2/3DS1), DX9 (KIR3DL1), UP-R1 (KIR2DL5). Gray histograms correspond to the isotype control.

To study the role of the CD94/NKG2C NKR in recognition of HCMV-infected cells, a reporter cell system was developed expressing the receptor segregated from other NKR. For this purpose, NKG2C and DAP12 were stably transduced in an available CD94-transfected human Jurkat leukemia T cell line. Jurkat-NKG2C+ cells were sorted and cloned by limiting dilution assessing the expression of adhesion molecules and NKR. A clone (97), which expressed CD94/NKG2C was selected (Figure 1A). These cells had downregulated CD3, displayed adhesion/co-stimulatory molecules (i.e., LFA-1, CD2, ICAM-1, and DNAM-1), but lacked activating (i.e., NKG2D, NKp46, NKp44, NKp30, CD16, aKIR) and inhibitory (i.e., iKIR, NKG2A, TIGIT, ILT2) NKR (Figure 1B). The marginal expression of ILT2 was particularly important as this HLA-I specific inhibitory receptor, which interacts with the UL18 HCMV molecules, was detectable in the parental Jurkat-CD94+ cells (not shown).

### Specific Recognition of HLA-E by Jurkat-NKG2C+ Reporter Cells

**Figure 2:**
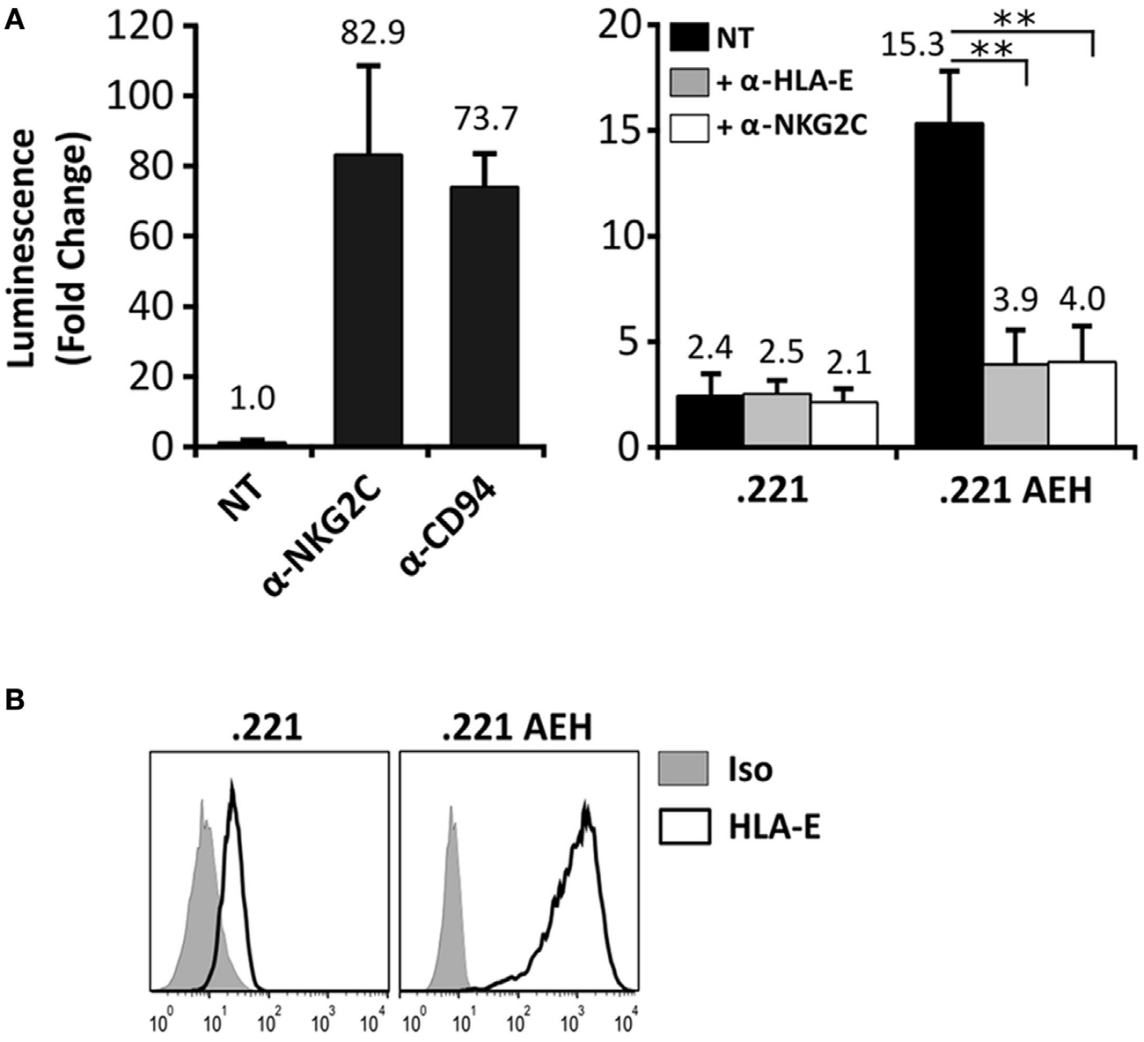
Specific engagement of the CD94/NKG2C receptor by HLA-E activates the Jurkat-NKG2C+ reporter. **(A)** Jurkat-NKG2C+ cells were electroporated with 3× NFAT/AP1-Luc plasmid, followed by stimulation with: (a) anti-NKG2C or anti-CD94 monoclonal antibodies (mAbs) pre-adsorbed to culture plates or (b) with .221 or .221-AEH cells. After 18–24 h, cells were collected, lysed, and Luc activity was measured. Blocking experiments were carried out pretreating .221 and .221-AEH cells with anti-HLA-E 3D12 mAb (gray bars) or Jurkat-NKG2C+ cells with anti-NKG2C mAb (white bars); NT, not treated. Data correspond to three independent experiments (mean ± SD). **(B)** HLA-E expression by .221 and .221-AEH was assessed prior to coculture with Jurkat-NKG2C+ cells (gray histograms represent the isotype control). Statistically significant differences are indicated (**p < 0.01).

**Figure 3:**
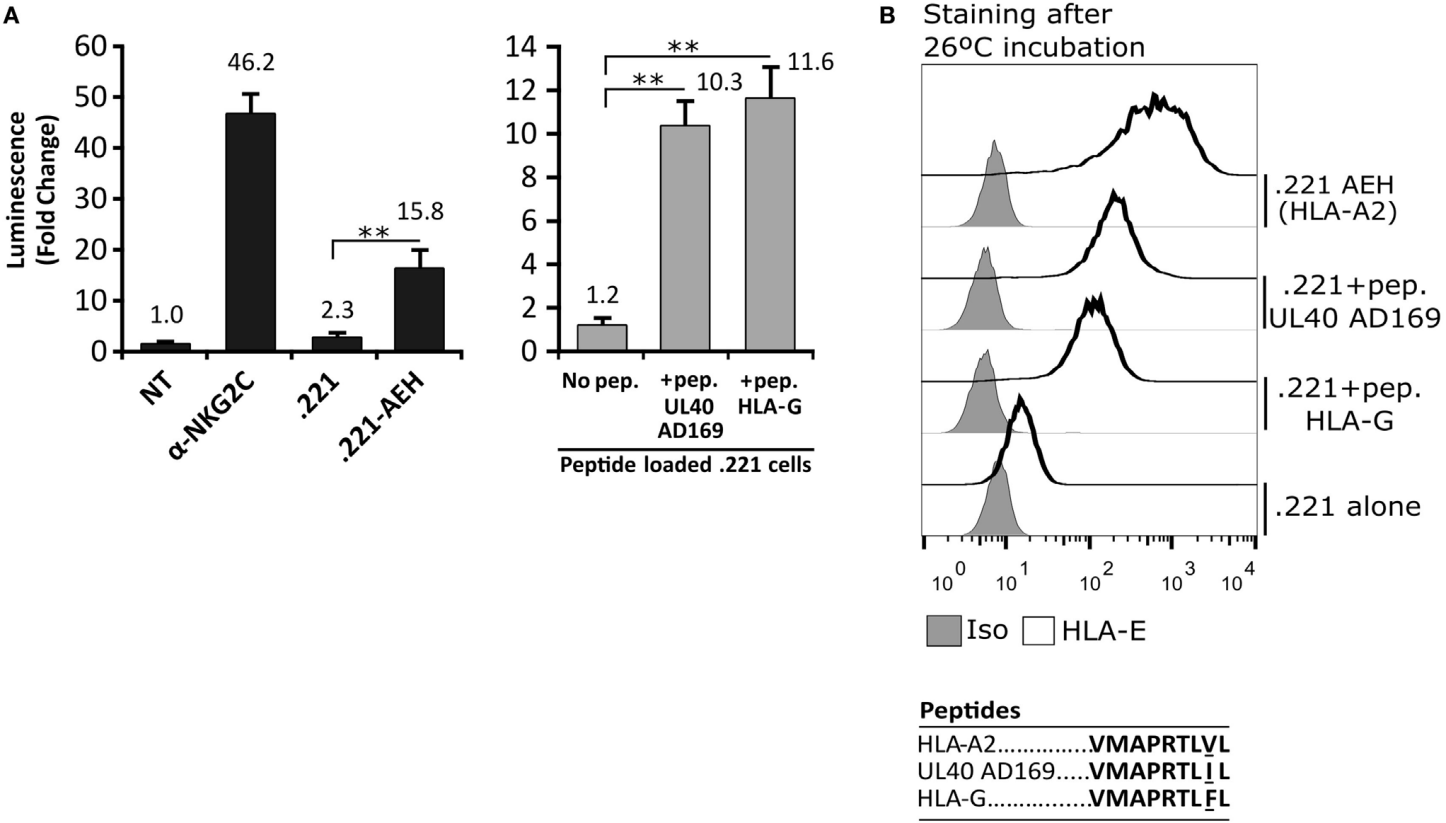
Activation of Jurkat-NKG2C+ cells by HLA-E loaded with the canonical human cytomegalovirus UL40 leader sequence-derived synthetic peptide on 721.221 cells. **(A)** As described for Figure 2, Jurkat-NKG2C+ cells were: (a) untreated (NT), (b) stimulated with plate coated anti-NKG2C, or (c) cocultured with the .221 or .221-AEH cells (black bars). In parallel, the response of Jurkat-NKG2C+ cells was assessed coculturing them with .221 cells preincubated at 26°C overnight with synthetic peptides corresponding to HLA-G (VMAPRTLFL) and AD169 UL40 (VMAPRTLIL) leader sequences, reported to stabilize HLA-E expression (gray bars). Data correspond to three independent experiments (mean ± SD). **(B)** HLA-E expression by .221-AEH and .221 cells preincubated with peptides prior to coculture with Jurkat-NKG2C+ cells; gray histograms represent the isotype control. Statistically significant differences are indicated (**p < 0.01).

In order to detect signaling by the CD94/NKG2C-DAP12 complex, Jurkat-NKG2C+ cells were transiently transfected with a plasmid encoding for Luciferase (Luc) under the control of NFAT/AP1-dependent promoter. Engagement of the receptor by solid-phase bound CD94- or NKG2C-specific mAbs triggered Luc expression (Figure 2A). Of note, a slightly higher background in Jurkat-NKG2C+ cells compared to the parental cell line suggested that the low constitutive HLA-E expression in Jurkat-NKG2C+ cells promoted a limited self-activation of the reporter (Figure S1 in Supplementary material). However, coculture of Jurkat-NKG2C+ cells with the .221-AEH cell line, which displays HLA-E in the absence of classical HLA-I, induced Luc expression and the response was specifically antagonized by soluble NKG2C- and HLA-E-specific mAbs (Figures 2A,B). Similar results were obtained stimulating Jurkat-NKG2C+ cells with the .221 cell line preincubated with synthetic HLA-I leader sequence peptides, known to stabilize surface HLA-E expression (Figures 3A,B). These results validated the sensitivity and specificity of the reporter system to detect CD94/NKG2C receptor–ligand interaction.

### Response of Jurkat-NKG2C+ Reporter Cells to Fibroblasts Infected by HCMV Laboratory Strains

**Figure 4:**
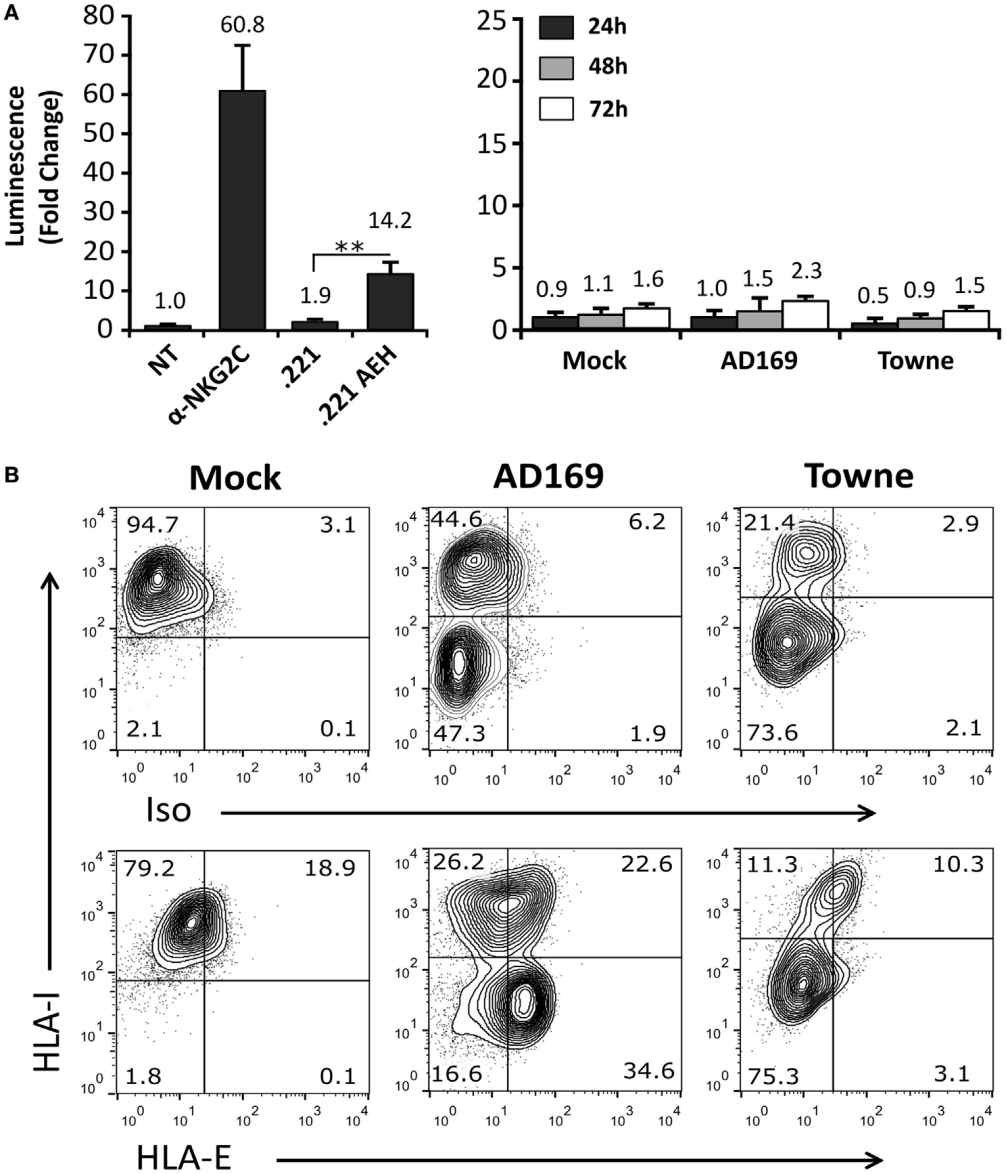
Cells infected with AD169 or Towne human cytomegalovirus (HCMV) strains do not activate the Jurkat-NKG2C+ reporter independently of HLA-E expression. **(A)** HFFF cells infected with AD169 or Towne HCMV strains (multiplicity of infection = 10) were cocultured at 24, 48, and 72 h postinfection with Jurkat-NKG2C+ cells electroporated with the reporter plasmid. The average infection rate for each experiment was >50%. As a control, reporter activation following CD94/NKG2C receptor engagement by specific monoclonal antibodies or HLA-E was assessed in parallel, as described in Figure 2. Data correspond to three independent experiments (mean ± SD). **(B)** Total HLA-I and HLA-E expression at 72 h postinfection in mock-treated and HCMV-infected HFFF cells. Statistically significant differences are indicated (**p < 0.01).

On that ground, experiments were carried out incubating Jurkat-NKG2C+ cells with HFFF, infected at different time-points (24–72 h) with HCMV laboratory strains (AD169 and Towne). As compared to positive controls stimulated with anti-NKG2C mAb or .221-AEH cells, no differences were perceived comparing Luc expression in Jurkat-NKG2C+ cocultured either with mock- or HCMV-infected HFFF (Figure 4A); similar results were obtained incubating the reporter with MRC5 fibroblasts (not shown). Total HLA-I expression was downregulated in cells infected by both HCMV strains. By contrast, HLA-E was preserved by AD169, which displays a canonical UL40 leader peptide (VMAPRTLIL) binding to the HLA class Ib molecule, but was lost in Towne-infected cells (Figure 4B). As reported (44) and according to the annotated GenBank sequences, this HCMV strain contains a deletion spanning the UL40 leader sequence. These results point out that the undetectable response of Jurkat-NKG2C+ to fibroblasts infected by laboratory strains was unrelated to their ability to sustain surface HLA-E expression.

### Response of Jurkat-NKG2C+ Reporter Cells to Fibroblasts Infected by HCMV Clinical Isolates

**Figure 5:**
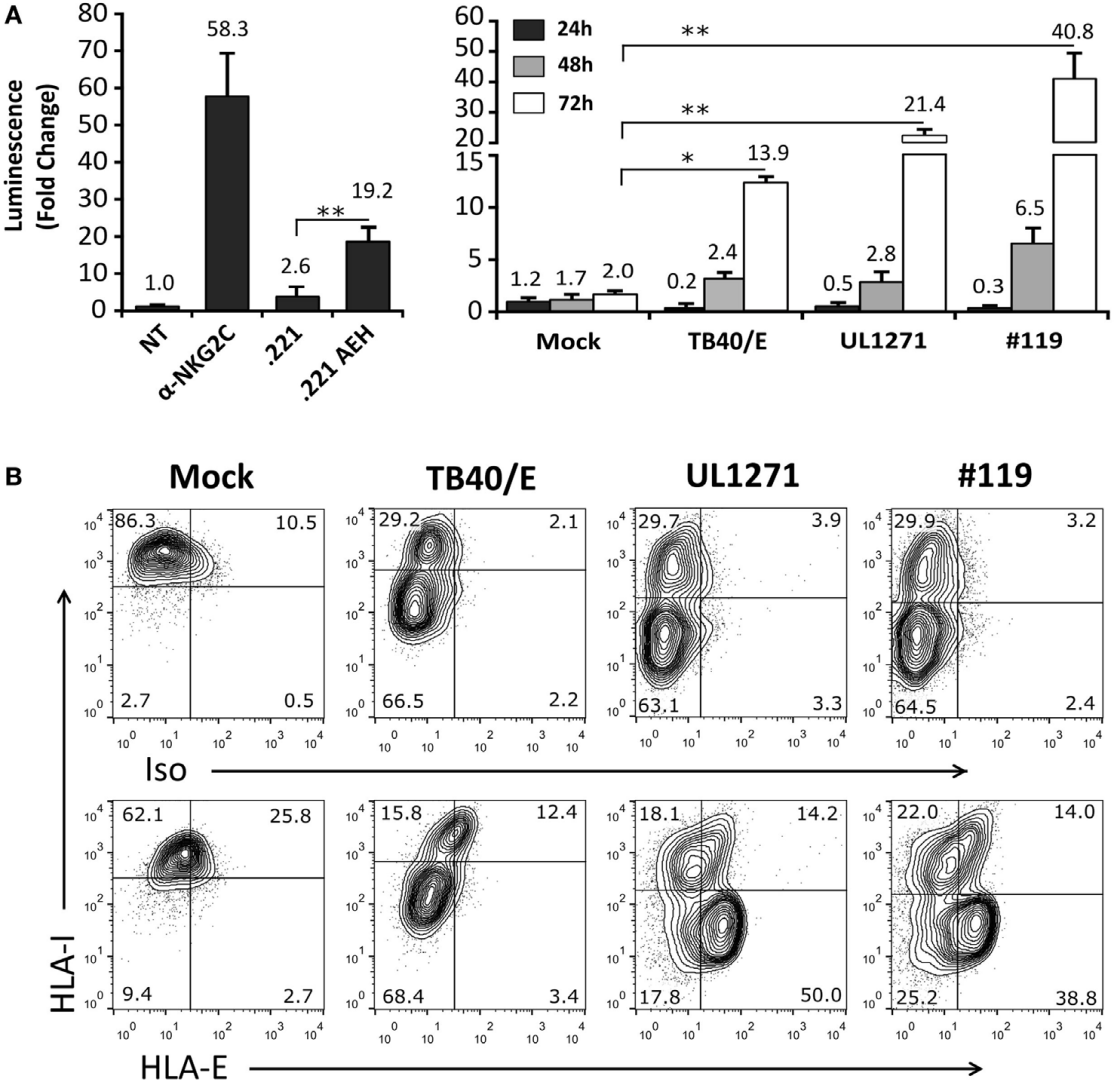
Activation of Jurkat-NKG2C+ reporter cells by HFFF infected with human cytomegalovirus TB40/E or clinical isolates is independent of HLA-E expression. **(A)** The Jurkat-NKG2C+ reporter was cocultured with HFFF cells at different time-points (24–72 h) postinfection with TB40/E or two different clinical isolates (UL1271 and #119) (multiplicity of infection = 10). Data correspond to three independent experiments (mean ± SD). Average infection rate for each experiment was >50%. **(B)** Total HLA-I and HLA-E surface expression was monitored at 72 h postinfection. Statistically significant differences are indicated (*p < 0.05; **p < 0.01).

**Figure 6:**
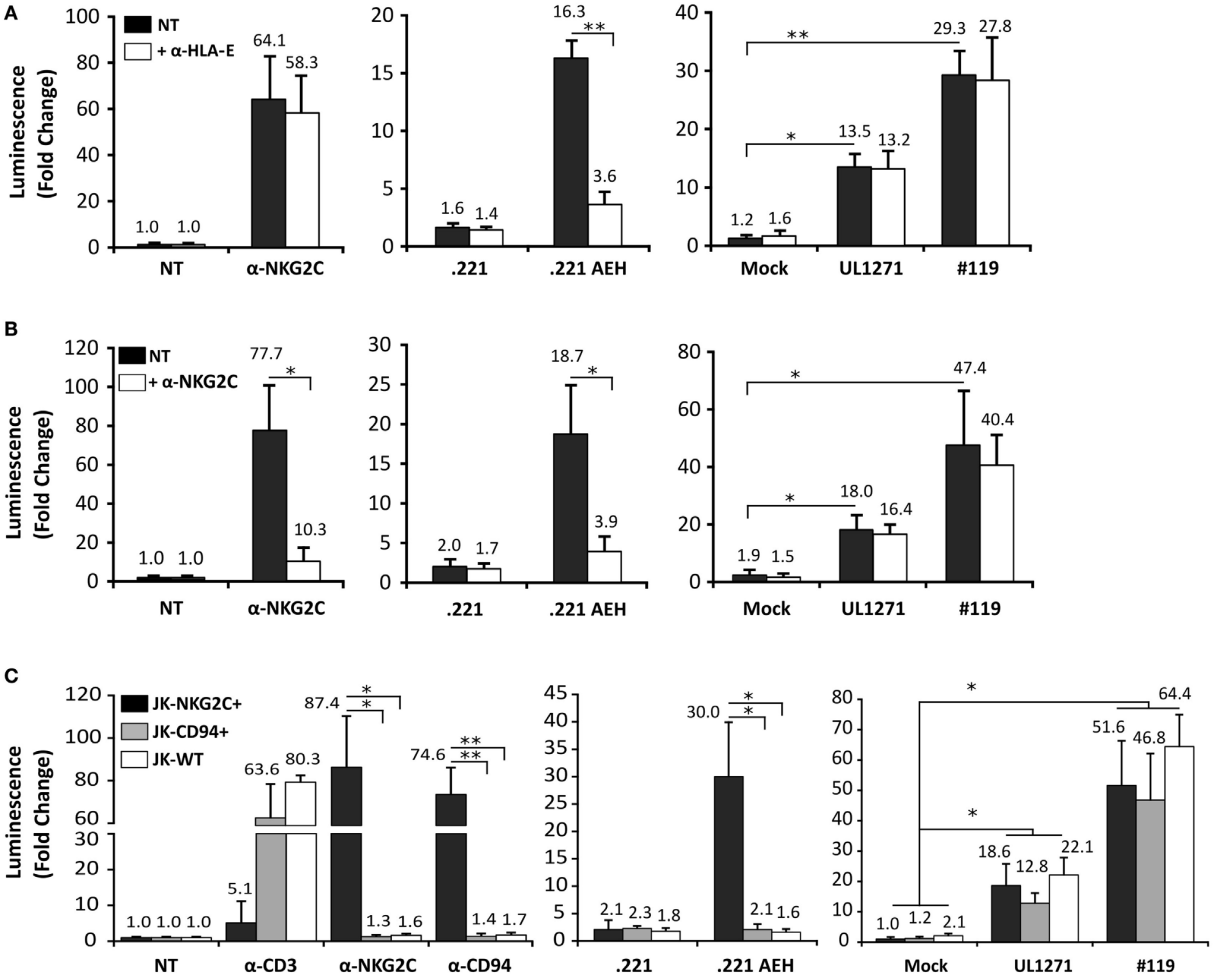
Jurkat-NKG2C+ activation by cells infected with human cytomegalovirus (HCMV) clinical isolates is independent of the CD94/NKG2C receptor. **(A)** Response of Jurkat-NKG2C+ reporter to HFFF infected by HCMV clinical isolates or .221-AEH cells preincubated with the 3D12 anti-HLA-E monoclonal antibody (mAb) (5 µg/ml). **(B)** The blocking effect of an anti-NKG2C mAb (clone MAB1381) (5 µg/ml) on the response of Jurkat-NKG2C+ cells to stimulation with either plate-bound anti-NKG2C mAb, .221-AEH cells or HFFF infected by HCMV clinical isolates (white bars) was assessed in parallel. Data correspond to three independent experiments (mean ± SD); the average infection rate for each experiment was >50%. **(C)** Jurkat-NKG2C+ (black bars), Jurkat-CD94+ (gray bars), and wild-type Jurkat (WT) (white bars) were stimulated with 1 µg/ml of plate-bound anti-CD3, -NKG2C, -CD94 mAbs, or cocultured with .221 or .221-AEH cells (negative and positive controls). Confluent HFFF cells infected with UL1271 and #119 clinical isolates (multiplicity of infection = 10) for 72 h were cocultured with the different Jurkat cell lines (NKG2C+, CD94+, and WT) previously electroporated with the reporter plasmid. Data correspond to three independent experiments (mean ± SD); the average infection rate for each experiment was >50%. Statistically significant differences are indicated (*p < 0.05; **p < 0.01).

TB40/E HCMV strain retains the ability to replicate in endothelial and myelomonocytic cells, differing from AD169 and Towne (45). Moreover, it is well established that HCMV clinical isolates substantially diverge from laboratory strains, rapidly undergoing important genomic changes upon *in vitro* passage, as reviewed in Ref. (46). Thus, additional experiments were carried out coculturing Jurkat-NKG2C+ cells with HFFF infected with TB40/E, which encodes for a UL40 leader peptide variant unable to preserve HLA-E surface expression (VVAPRTLIL) (25), or with two HCMV clinical isolates (#UL1271 and #119), which share the canonical UL40-nonamer and preserved HLA-E expression (Figures 5A,B). Remarkably, a specific response of the reporter was detected upon infection with these viruses, which appeared greater for #119 and gradually increased along postinfection time. Yet, controls indicated that Luc expression in response to HFFF infected with HCMV clinical isolates was not inhibited by pretreatment with anti-NKG2C or -HLA-E mAbs (Figures 6A,B); similar results were obtained with anti-CD94 F(ab′)_2_ fragments (data not shown), ruling out the potential influence of an overlapping agonistic effect mediated by anti-NKG2C bound to virus-encoded FcR. Moreover, the effect was also induced in CD94+ and wild-type Jurkat cells, which lacked NKG2C, unequivocally revealing that reporter activation was independent of this NKR (Figure 6C); similar results were obtained with TB40/E-infected cells (data not shown). As this response was dominant, the possibility that it might eventually mask a subtle involvement of CD94/NKG2C cannot be entirely ruled out.

## Discussion

A number of observations indirectly support the hypothesis that a specific and direct interaction of CD94/NKG2C with infected cells contributes to drive the adaptive NK cell response; yet, molecular evidence remains thus far elusive (10). To explore the presence of putative ligand(s) for CD94/NKG2C in HCMV-infected cells, the receptor was stably expressed along with DAP12 in the human Jurkat leukemia cell line, transiently transfected with an NFAT/AP1-dependent Luc-encoding reporter. The rationale for using a human parental cell line was based on our previous experience with heterologous CD94/NKG2C+ rat basophilic leukemia cells (RBL), which failed to respond to receptor engagement by HLA-E+ .221-AEH cells (not shown). A Jurkat-NKG2C+ clone that shared with NK cells key adhesion receptors (i.e., LFA-1, CD2, and DNAM1) but lacked inhibitory NKR, was selected.

The possibility that constitutive HLA-E expression by Jurkat-NKG2C+ cells might promote self-activation of the reporter, potentially impairing its responsiveness to external stimuli, was beforehand considered a potential drawback. Yet, Luc expression in Jurkat-NKG2C+ cells was induced following stimulation with CD94 or NKG2C-specific mAbs. Importantly, reporter activation was detectable upon interaction with HLA-E+ .221-AEH cells, as well as with .221 cells incubated with synthetic peptides which stabilized surface expression of endogenous HLA-E; in these settings, inhibition by anti-NKG2C and -HLA-E mAbs supported specific receptor-ligand engagement. By contrast, reporter activation was undetectable upon interaction of Jurkat-NKG2C+ cells with HFFF or MRC5 fibroblasts following infection with common HCMV laboratory strains (i.e., AD169, Towne), regardless of their ability to preserve surface HLA-E expression in infected cells.

Among variables that may potentially condition the expression of CD94/NKG2C ligand(s) by HCMV-infected cells, genomic differences between viral strains and changes associated to *in vitro* passage of the virus were considered potentially important. In fact, cells infected with two different clinical isolates and, to a lesser degree, with the endotheliotropic TB40/E strain, triggered Jurkat-NKG2C+ activation. Yet, this response was not inhibited by NKG2C- nor HLA-E-specific blocking mAbs and, furthermore, was independent of CD94/NKG2C expression. Experiments were carried out to explore the molecular basis of this dominant effect, which might potentially mask CD94/NKG2C-specific signaling. Pretreatment of Jurkat-NKG2C+ with a calcineurin inhibitor (FK506) hampered Luc expression triggered by anti NKG2C mAb or .221-AEH cells, but did not prevent the response of Jurkat-NKG2C+ cells to HCMV clinical isolates (data not shown), pointing out that a non-conventional activation of the reporter plasmid was induced under these experimental conditions. Further studies are required to assess the possibility of controlling this drawback, eventually re-designing the system.

NKG2C+ NK cells were reported to be activated upon interaction with primary aortic endothelial cells infected by the VHL/E HCMV strain (47); nevertheless, no data supporting an involvement of the receptor (e.g., blocking with anti-CD94 or -NKG2C mAbs) were provided. In our hands, Jurkat-NKG2C+ did not detectably respond to HCMV-infected MRC5 fibroblasts nor to human umbilical vein endothelial cells. Yet, the possibility that expression of putative CD94/NKG2C ligands might vary depending on the infected cell lineage is not ruled out.

Our observations should be discussed in the framework of previous indications supporting a CD94/NKG2C receptor interaction with infected cells. In this regard, NKG2C+ NK cells were shown to undergo *in vitro* expansion upon coculture with AD169 or Towne-infected fibroblasts (22, 23). The effect was dependent on cytokines (i.e., IL-12, IL-15) and was prevented by blocking anti-CD94, -NKG2C, or -HLA-E mAbs. The response was observed only in samples from some HCMV+ blood donors displaying baseline NKG2C^bright^ NK cell expansions, indicating that these experimental conditions promoted their proliferation, but did not strictly reproduce the differentiation process induced *in vivo* by primary HCMV infection. Of note, *in vitro* expansion was prevented upon infection by an HCMV BACmid mutant lacking the whole set of viral genes (i.e., US2-US11), which target HLA-I molecules, thus preserving their expression in infected cells (22). These experiments were interpreted as an indication of a relatively weak stimulation *via* CD94/NKG2C that was overridden by inhibitory KIR-HLA-I interactions which, by contrast, did not impair CD16-triggered NK cell effector functions against cells infected by the same virus (28).

On the other hand, a relation of NKG2C gene copy number with the magnitude of NKG2C+ NK cell expansion has been reported in HCMV+ healthy blood donors, in children with congenital infection and in renal transplant recipients (9, 14, 32, 48). Moreover, CD94/NKG2C surface expression levels and activation upon engagement by HLA-E were also greater in NKG2C^bright^ NK cells from *NKG2C*^*+/+*^ than *NKG2C*^*+/del*^ individuals (9). These data supported that subtle differences in CD94/NKG2C surface density may quantitatively influence adaptive NK cell differentiation/activation in response to HCMV, thus providing another indirect indication for a relatively low avidity of the hypothetical receptor–ligand interaction. The increased surface expression of CD94/NKG2C in differentiated adaptive NK cells (NKG2C^bright^) from HCMV+ subjects as compared to the profile detected in non-infected individuals (NKG2C^dim^) is also in line with this view. Of note, a limited affinity and/or reduced expression levels of the putative CD94/NKG2C ligand in infected cells may presumably render this pathway particularly sensitive to viral immune evasion mechanisms, eventually accounting for the undetectable activation of NKG2C+ NK cell effector functions and of Jurkat-NKG2C+ cells, in that case, independently of HLA-specific inhibitory receptors.

Despite the negative results obtained with the reporter system, the existence of specific ligand(s) for CD94/NKG2C in HCMV-infected cells still remains a plausible hypothesis, compatible with the involvement of a viral glycoprotein or/and an HLA-E-peptide complex. The latter option is suggested by the blocking effect of an anti-HLA-E mAb in the expansion of NKG2C+ NK cells cocultured with infected fibroblasts (23). A first candidate to be considered is the UL40 leader sequence-derived peptide reported to promote HLA-E expression in HCMV-infected cells by a mechanism refractory to the viral US6 TAP inhibitor (34) and favored by the intrinsic HLA-E resistance to other viral molecules targeting HLA-I (49). UL40-dependent HLA-E expression in infected cells was shown to inhibit NK cell activation by engagement of the CD94/NKG2A inhibitory receptor, presumably contributing to immune evasion (34). The canonical UL40-derived nonamer (VMAPRTLIL) binding to HLA-E is identical to that displayed by some HLA-I leader sequences, predictably promoting a low affinity interaction with CD94/NKG2C (50). Heatley et al. (51) compared the ability of a panel of UL40-nonamer variants to stabilize HLA-E in RMA-S cells, assessing the interaction of the corresponding HLA-E/peptide complexes with CD94/NKG2A or CD94/NKG2C by surface plasmon resonance assays, as well as their recognition by NK cell subsets, respectively, displaying these NKR. Of note, quantitative differences in the interaction of distinct HLA-E-peptide complexes were noticed, but none appeared preferentially recognized by CD94/NKG2C.

It is conceivable that HLA-E expression by HCMV-infected cells, in which HLA-I ligands for inhibitory KIRs are downregulated, might promote specific activation of NKG2C+ NKG2A- NK cells. Supporting this hypothesis, Jurkat-NKG2C+ cells were activated by .221 cells displaying HLA-E bound to the synthetic AD169 UL40-derived peptide (VMAPRTLIL), shared by some HLA-I alleles. Nevertheless, no reporter response was perceived following interaction with AD169-infected cells expressing HLA-E, pointing out to the influence of additional factors, and indicating that experimental approaches based on loading target cells with synthetic peptides provide valuable information but do not precisely reflect the complexity of HCMV infection. A putative role of UL40 in NKG2C+ NK cell expansion, detected following coculture with AD169-infected cells, was addressed employing a UL40 deletion mutant generated in an AD169 BACmid (HB5) (22, 23). In this setting, cells infected with ΔUL40 HB5 retained the ability to promote *in vitro* expansion of NKG2C+ NK cells; however, a caveat for interpreting these experiments is the gene deletion encompassing US2-US6 introduced for the generation of the HB5 BACmid. In fact, HCMV impairs surface expression of HLA-I molecules but not their biosynthesis and, therefore, endogenous HLA-I leader sequences in the absence of US6 may be presented by HLA-E in HB5-infected cells, eventually competing with the UL40-derived nonamer, or even replacing it in ΔUL40 HB5-infected HFFF. On the other hand, NKG2C+ NK cell expansions detected in cocultures with Towne-infected fibroblasts (22) may indirectly provide a case against the involvement of HLA-E expression, which was downregulated consistent with the existence of a genomic deletion spanning the UL40 leader sequence of this HCMV strain (44). Of note, no relation of the HLA-E dimorphism with the expansion of NKG2C+ cells was noticed in previous studies (7, 9).

In summary, molecular evidence supporting that a specific interaction of CD94/NKG2C with infected cells drives the adaptive NK cell response to HCMV remains elusive, leaving unanswered key interrelated questions, particularly, the cellular mechanisms underlying the stable expansion of NKG2C^bright^ NK cells and the basis for the wide variability of this effect in HCMV+ individuals. Observations in immunocompromised patients suggested that this pattern of response may compensate an inefficient T-cell-mediated control of the primary infection, eventually determined in healthy individuals by viral/host genetic factors as well as by other circumstantial variables (e.g., age at infection, viral load, etc.).

We hypothesize that primary HCMV infection promotes the differentiation, proliferation, and survival of a pool of progenitors, possibly stemming from NKG2C^dim^ NKG2A- NK cells present in seronegative individuals. Increased surface expression of CD94/NKG2C presumably constitutes a key early event, facilitating activation of this NK cell subset upon interaction with low-avidity ligand(s) displayed by infected cells. Such NKG2C-driven selection is dependent on cytokines required for adaptive NK cell differentiation/expansion and may be tuned by specific KIR-HLA-I interactions, consistent with the oligoclonal KIR expression profile of adaptive NK cells (8, 52). Following a contraction phase after viral replication is controlled, a pool of long-lived NKG2C^bright^ NK cells with clonal expansion potential survive, and homeostatic proliferation contributes to their persistent increased numbers in the circulation. The process is reminiscent of the generation of memory cytotoxic T lymphocytes as proposed for the response of Ly49H in mice (53); in fact, responsiveness of differentiated NKG2C^bright^ NK cells to cytokines (e.g., IL-15, IL-2) becomes dependent on signaling by activating NKR (e.g., NKG2C or CD16). HCMV reactivation/reinfection as well as other infectious pathogens may boost antibody- and cytokine-dependent activation of adaptive NK cells, leading to their progressive acquisition of late differentiation features (e.g., FcRγ downregulation). Further efforts are warranted to understand how HCMV infection resets the NK-cell compartment homeostasis in some individuals, and the implications that such persistent reconfiguration of the NK cell compartment may have in the development of the immune response under different pathological conditions.

## Author Contributions

AP developed the reporter system, carried out the experimental work, and revised the manuscript. MC-G collaborated in the experimental development. DF and AA collaborated in the genetic analysis of HCMV as well as in critical discussion of the results. HH contributed with essential support for the analysis of HCMV clinical isolates as well as in critical discussion of the results. AM contributed to the design, follow-up, and interpretation of the results. ML-B proposed the experimental approach, contributed to the design, follow-up, and interpretation of results, and wrote the draft that was revised by all authors.

## Conflict of Interest Statement

Authors individually declare that the research was conducted in the absence of any commercial or financial relationship that could be construed as a potential conflict of interest. The reviewer CF and handling editor declared their shared affiliation.
