# Supplementary material for: Elusive Role of the CD94/NKG2C NK Cell Receptor in the Response to Cytomegalovirus: Novel Experimental Observations in a Reporter Cell System
Source: Front Immunol. 2017 Oct 24;8:1317. doi: 10.3389/fimmu.2017.01317 (PMC5660692; doi:10.3389/fimmu.2017.01317)
Supplement: Supplementary file 1 [file image_1.pdf]

A

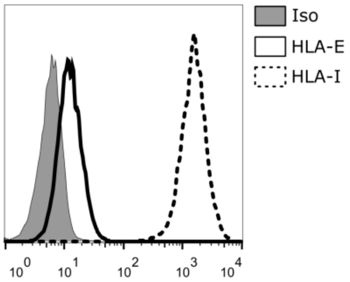

B

CONTROLS	*Raw Luminescence Unit (RLU)*					
	NT	***α-CD3***	***α-NKG2C***	***α-CD94***	***.221***	***.221 AEH***
**JK-NKG2C+**	2246	2506	135355	148521	1623	38540
**JK-CD94+**	1344	107221	2015	2684	2820	2916
**JK-WT**	1017	85210	1624	2159	2010	1988

C

CONTROLS	*Fold Change (F.C.)*					
	NT	***α-CD3***	***α-NKG2C***	***α-CD94***	***.221***	***.221 AEH***
**JK-NKG2C+**	1,0	1,1	60,3	66,1	0,7	17,2
**JK-CD94+**	1,0	79,8	1,5	2,0	2,1	2,2
**JK-WT**	1,0	83,8	1,6	2,1	2,0	2,0

D

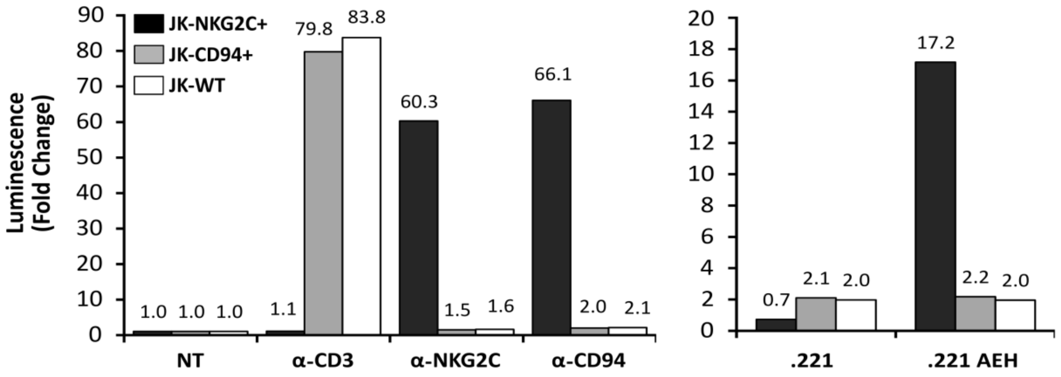

Supplementary Figure 1: Induction of Luciferase activity in Jurkat-NKG2C+, Jurkat-CD94+ and wild-type Jurkat (JK-WT) cells.

All three cell lines were electroporated with 3X NFAT/AP1-Luc plasmid, followed by stimulation with anti-CD3, anti-NKG2C or anti-CD94 mAbs pre-adsorbed to culture plates or, alternatively, with the .221 or .221-AEH cell lines. After 18-24h cells were collected, lysed and Luc activity was measured. **A**) Histogram displaying HLA-E (solid line) and total HLA class I expression (dashed line) in Jurkat-NKG2C clone 97. **B)** Table including raw luciferase counts as measured in each condition. **C and D**) Table and bar graphs showing data normalized to the respective non-treated (NT) Jurkat cell line and represented as fold-change induction.
